# Impact of osteoarthritis on activities of daily living: does joint site matter?

**DOI:** 10.1007/s40520-019-01163-0

**Published:** 2019-03-21

**Authors:** Michael A. Clynes, Karen A. Jameson, Mark H. Edwards, Cyrus Cooper, Elaine M. Dennison

**Affiliations:** 1 MRC Lifecourse Epidemiology Unit, Southampton General Hospital, University of Southampton, Southampton, SO16 6YD UK; 2 National Institute for Health Research Musculoskeletal Biomedical Research Unit, University of Oxford, Oxford, OX3 7LE UK

**Keywords:** Osteoarthritis, Activities of daily living, Knee, Hip

## Abstract

**Background:**

We consider the relationships between a clinical and radiological diagnosis of knee or hip OA and activities of daily-living (ADL) in older adults.

**Methods:**

Data were available for 222 men and 221 women from the Hertfordshire Cohort Study (HCS) who also participated in the UK component of the European Project on Osteoarthritis (EPOSA). Participants completed the EuroQoL survey where they reported if they had difficulties with mobility, self-care, usual activities and movement around their house. Hip and knee radiographs were graded for overall Kellgren and Lawrence score (positive definition defined as a 2 or above). Clinical OA was defined using American College of Rheumatology criteria.

**Results:**

In men, a clinical diagnosis of hip or knee OA were both associated with reported difficulties in mobility, ability to self-care and performing usual-activities (hip OA: OR 17.6, 95% CI 2.07, 149, *p* = 0.009; OR 12.5, 95% CI 2.51, 62.3, *p* = 0.002; OR 4.92, 95% CI 1.06, 22.8, *p* = 0.042 respectively. Knee OA: OR 8.18, 95% CI 3.32, 20.2, *p* < 0.001; OR 4.29, 95% CI 1.34, 13.7, *p* = 0.014; OR 5.32, 95% CI 2.26, 12.5, *p* < 0.001 respectively). Similar relationships were seen in women, where in addition, a radiological diagnosis of knee OA was associated with difficulties performing usual activities (OR 3.25, 95% CI 1.61, 6.54, *p* = 0.001). In general, men with OA reported stronger associations between moving around the house, specifically around the kitchen (clinical hip OA: OR 13.7, 95% CI 2.20, 85.6, *p* = 0.005; clinical knee OA OR 8.45, 95% CI 1.97, 36.2, *p* = 0.004) than women.

**Discussion and conclusion:**

Clinical OA is strongly related to the ability to undertake ADL in older adults and should be considered in clinic consultations when seeing patients with OA.

## Introduction

The increase in life expectancy and the subsequent ageing population has led to a higher prevalence of chronic, non-communicable diseases and in particular musculoskeletal (MSK) disorders. After cardiovascular diseases, malignant neoplasms and chronic respiratory diseases, MSK disorders are the fourth leading cause of morbidity in older people [1]. Osteoarthritis (OA) is the most common of the MSK disorders affecting older people [2]. It has been estimated that OA affects over 26 million people in the USA, and around 1.6–3.4 million in England and Wales [3, 4]. There is a significant economic burden associated with OA, largely secondary to the effects of disability associated with OA, comorbid diseases and cost of treatment [4].

In OA there is degeneration of the joints involving the articular cartilage and many of the surrounding tissues [5]. There is a breakdown of the equilibrium between breakdown and repair of joint tissue, leading to the loss of articular cartilage, remodelling of subchondral bone, osteophyte formation, ligament laxity, periarticular muscle weakening, and occasionally synovitis [6]. This can occur in any joint, but the joints more commonly afflicted by OA are the hands, feet, facet joints and large weight-bearing joints, such as the knees and hips [5]. Joint degeneration in OA results in pain, which in turn leads to stiffness and restricted movement.

Epidemiological studies of OA have principally defined OA using two methods: radiographic and clinical [7, 8]. A radiographic definition of OA captures the structural changes in the joints of interest. The majority of studies employ the radiographic technique first proposed by Kellgren and Lawrence [9], which characterises knee OA into five grades (0, normal to 4, severe) with a score of 2 or above representing OA. A radiological diagnosis of OA alone, however, may not accurately reflect the clinical burden of the disease as studies have shown that pain in OA is heightened by co-morbid illness, muscle-strength, mood, cognition and disability [10]. An alternative method of defining OA is to utilise clinical criteria. In the early 1990s, the American Rheumatism Association (ACR) developed a definition of OA that takes into account medical history, laboratory test results and physical examinations.

OA can contribute to inactivity with ageing, secondary to pain and reduced function, thus ultimately impairing quality of life. It is well established that OA pain, swelling or stiffness can make it difficult for individuals to perform simple activities of daily living (ADL) such as opening boxes of food, tucking in bedsheets, writing, using a computer mouse, driving a car, walking, climbing stairs and lifting objects [11] but to our knowledge the impact the condition has on everyday function has been little studied in individuals who are not awaiting joint replacement surgery.

The EuroQol survey is a standardized instrument for measuring generic health status developed in 1990 by the EuroQol group which is a multidisciplinary team of researchers from five European countries; The Netherlands, UK, Sweden, Finland, and Norway [12]. Their aim was to develop an instrument which is not specific to disease but standardized and can be used as a complement for existing health-related quality of life (HRQoL) measures. In the current study, we use components of the Euroqol survey to consider the relationships between a clinical or radiological diagnoses of lower limb OA and ADL in older men and women.

## Methods

The study participants were 222 men and 221 women from the Hertfordshire Cohort Study (HCS) who also participated in the UK component of the European Project on Osteoarthritis (EPOSA). The Hertfordshire Cohort Study (HCS) is a population-based UK cohort of older adults. Study design and recruitment have been described in detail previously [13]. In brief, we traced men and women born between 1931 and 1939 in Hertfordshire and who still lived there in 1998–2003. A nurse-administered questionnaire, which included details of socioeconomic status and dietary calcium intake, was conducted at this time. In a follow-up study in 2011–2012, 443 participants consented to a home visit by a trained research nurse. At this visit a nurse-administered questionnaire was again administered which included details of smoking status, alcohol consumption and physical activity (average minutes per day spent walking, cycling, gardening, playing sport and doing housework in the last 2 weeks). Height was measured to the nearest 0.1 cm and weight to the nearest 0.1 kg on a SECA floor scale (Chasmors Ltd, London, UK). Body mass index (BMI) was calculated as weight divided by height^2^ (kg/m^2^). Participants also answered questions taken from the EuroQol study where they were asked: “Do you have problems with mobility?”; “Do you have problems with self-care?”; “Do you have problems undertaking your usual activities?” [12]. Participants were then asked more detailed questions on mobility where they were asked: “Do you have problems moving around inside and outside your house?”; “Do you have problems moving around your bathroom?”; “Do you have problems moving around your kitchen?”; “Do you have problems moving around your toilet?”; “Do you have problems accessing public facilities such as grocery shops, bus stops or banks?”. Radiographs were taken of the hip and knees under standardised conditions at a local hospital after the home visit. Clinical OA was defined based on algorithms developed by the American College of Rheumatology [14].

A clinical diagnosis of hip OA was made if pain, as assessed by WOMAC, was present in addition to all of the following: (1) pain associated with hip internal rotation in at least one side; (2) morning stiffness lasting < 60 min evaluated by the WOMAC stiffness subscale (score from ‘mild’ to ‘extreme’); and (3) age of over 50 years [15]. Pain was assessed using the Western Ontario and McMaster Universities OA Index (WOMAC) pain subscale score. The WOMAC is a 24-item questionnaire with three subscales measuring pain (five items), stiffness (two items), and physical function (17 items) [16].

To diagnose clinical knee OA the patient had to experience knee pain and any three of the following: (1) bony tenderness in at least one side on examination; (2) crepitus on active motion in at least one side on examination; (3) less than 30 min of morning stiffness, evaluated by the WOMAC stiffness subscale; (4) no palpable warmth of synovium in both knees on examination; (5) age over 50 years; or (6) bony enlargement in at least one side on examination.

Radiographs were graded according to Kellgren and Lawrence (KL). KL classifies OA into five grades (0, normal to 4, severe). The KL grading system is briefly described as follows: grade 0—no radiographic features of OA are present; grade 1—unlikely narrowing of the joint space and possible osteophytes on the radiograph; grade 2—small osteophytes and possible narrowing of the joint space; grade 3—multiple, moderately sized osteophytes, definite joint space narrowing, some sclerotic areas and possible deformation of bone ends; and grade 4—multiple large osteophytes, severe joint space narrowing, marked sclerosis and definite bony end deformity [9]. In our study, a positive definition of OA reflected a KL score of 2 or above. The radiographs were all graded by two experienced rheumatologists with good inter-observer agreement.

Stata version 14 was used for all analyses. Study participants’ characteristics were summarised using means and standard deviations (SD) or medians and interquartile ranges (IQR) for continuous variables, and numbers and percentages for binary and categorical variables. Logistic regression was used to model the association between self-reported OA, clinical OA and radiographic OA with the components of the EuroQol survey and questions on mobility. These analyses were completed with and without adjustment for age, BMI, social class, activity, alcohol intake, baseline dietary calcium and smoking status and years since menopause and HRT use in women. These confounders were selected as they have been shown to be associated with the ability to undertake ADL and OA in previous studies. A study by Pollard and colleagues on a cohort of 763 people who had been diagnosed with OA in Somerset and Avon, UK showed that impact of OA on ADL appears to vary with respect to social deprivation [17]. A recent study by Magnusson and colleagues showed alcohol was associated with inflammatory hand OA whereas smoking appeared to be protective [18]. Farr et al. have demonstrated using accelerometry that the majority of patients with knee OA do not meet the recommended levels of physical activity [19] which results in weight gain and obesity, progression of OA and impairment of function [20]. Finally, significantly higher concentrations of calcium have been found in the meniscus of individuals with knee OA undergoing total knee replacement surgery [21].

## Results

The mean [standard deviation (SD)] age of study participants was 75.5 (2.5) and 75.8 (2.6) years in men and women, respectively. The mean body mass index (BMI) was 27.9 kg/m^2^ (SD 3.9) in men and 28.4 kg/m^2^ (SD 5.1) in women. Men had a lower median activity time than women in the last 2 weeks [176 min/day (IQR 105–270) and 200 min/day (IQR 135–283) respectively], although this did not reach statistical significance (*p* = 0.089). A higher proportion of men were current smokers [5% (*n* = 11) vs 2.7% (*n* = 6) of women] (Table 1).

**Table 1:** Participant demographics

	Men		Women	
	*n*	Mean (SD)	*n*	Mean (SD)
Age (years)	222	75.5 (2.5)	221	75.8 (2.6)
Height (cm)	221	172.7 (6.5)	217	158.8 (6.1)
BMI (kg/m^2^)	221	27.9 (3.9)	217	28.4 (5.1)

	*n*	Median (IQR)	*n*	Median (IQR)
Activity time in last 2 weeks (min/day)	205	176 (105–270)	200	200 (135–283)
Daily dietary calcium intake (mg)	222	1221 (1021–1432)	221	1105 (939–1281)
Alcohol consumption (units/week)	222	6.5 (1.0–14.0)	221	0.5 (0.0–3.5)

	Total *n*	*n* (%)	Total *n*	*n*%
Clinical OA				
Hip	219	7 (3.2)	216	13 (6.0)
Knee	216	26 (12.0)	216	41 (19.0)
Radiographic OA				
Hip	201	93 (46.3)	192	78 (40.6)
Knee	201	101 (50.2)	201	118 (58.8)

Seven (3.2%) men and 13 (6.0%) women had a clinical diagnosis of hip OA. Radiographic hip OA was more common, affecting 46.3% (*n* = 93) of men and 40.6% (*n* = 78) of women. Knee OA was overall more common than hip OA in both sexes with the radiographic diagnosis again being more prevalent [50.2% (*n* = 101) of men and 58.7% (*n* = 118) of women], compared with the clinical diagnosis [12% (*n* = 26) of men and 19% (*n* = 41) of women] (Table 1).

In men, a clinical diagnosis of hip or of knee OA were both associated with reported difficulties in mobility, ability to self-care and performing usual activities (hip OA: OR 17.6, 95% CI 2.07, 149, *p* = 0.009; OR 12.5, 95% CI 2.51, 62.3, *p* = 0.002; OR 4.92, 95% CI 1.06, 22.8, *p* = 0.042, respectively. Knee OA: OR 8.18, 95% CI 3.32, 20.2, *p* < 0.001; OR 4.29, 95% CI 1.34, 13.7, *p* = 0.014; OR 5.32, 95% CI 2.26, 12.5, *p* < 0.001 respectively). With the exception of the association between clinical knee OA and self-care these findings remained robust following adjustment for confounders (Table 2). Very similar relationships were seen in women, where clinical OA at hip and knee were both associated with reported difficulties in mobility, ability to self-care and performing usual activities (Hip OA: OR 5.49, 95% CI 1.63, 18.5, *p* = 0.006; OR 8.81, 95% CI 2.67, 29.0, *p* < 0.001 and OR 15.9, 95% CI 3.40, 74.0, *p* < 0.001 respectively; Knee OA: OR 7.51, 95% CI 3.56, 15.9, *p* < 0.001; OR 9.52, 95% CI 3.87, 23.4, *p* < 0.001 and OR 9.20, 95% CI 4.31, 19.7, *p* < 0.001, respectively). The association between clinical knee OA and difficulties with mobility, self-care and performing usual activates remained robust following adjustment for confounders but for clinical hip OA only the association with problems performing usual activities remained significant following adjustment for confounders (OR 19.6, 95% CI 1.18, 326, *p* < 0.038). Additionally, in women a radiological diagnosis of knee OA was associated with similar, though less marked, reported difficulties in mobility, self-care and performing usual activities (OR 2.56, 95% CI 1.36, 4.85, *p* = 0.004; OR 2.81, 95% CI 1.00, 7.90, *p* = 0.050 and OR 3.25, 95% CI 1.61, 6.54, *p* = 0.001 respectively). Aside from the association with self-care these associations remained robust following adjustment for confounders (Table 3).

**Table 2:** Hip and knee OA as explanatory variables for EuroQoL scores in men

	Unadjusted				Adjusted^a^			
	*n*	OR	95% CI		*n*	OR	95% CI	*p* value
*EuroQoL mobility*								
Clinical hip OA	219	17.6	(2.07, 149)	0.009	192	250	(6.45, 9663)	0.003
Radiographic hip OA	201	0.94	(0.50, 1.78)	0.846	176	1.90	(0.83, 4.31)	0.127
Clinical knee OA	216	8.18	(3.32, 20.2)	< 0.001	188	7.81	(2.47, 24.8)	< 0.001
Radiographic knee OA	201	1.15	(0.61, 2.16)	0.661	175	0.60	(0.27, 1.32)	0.204
*EuroQoL self-care*								
Clinical hip OA	219	12.5	(2.51, 62.3)	0.002	192	43.9	(3.86, 499)	0.002
Radiographic hip OA	201	1.68	(0.51, 5.47)	0.392	176	2.96	(0.72, 12.1)	0.131
Clinical knee OA	216	4.29	(1.34, 13.7)	0.014	188	2.33	(0.56, 9.81)	0.247
Radiographic knee OA	201	3.16	(0.83, 12.1)	0.091	175	3.14	(0.60, 16.5)	0.176
*EuroQoL usual activities*								
Clinical hip OA	218	4.92	(1.06, 22.8)	0.042	191	10.7	(1.60, 71.3)	0.015
Radiographic hip OA	200	0.82	(0.41, 1.64)	0.571	175	1.16	(0.50, 2.66)	0.730
Clinical knee OA	215	5.32	(2.26, 12.5)	< 0.001	187	3.02	(1.07, 8.49)	0.036
Radiographic knee OA	200	1.33	(0.67, 2.65)	0.422	174	0.80	(0.35, 1.84)	0.605

^a^Adjusted for age, BMI, social class, smoker status, alcohol consumption, activity and baseline dietary calcium

**Table 3:** Hip and knee OA as explanatory variables for EuroQoL scores in women

	Unadjusted				Adjusted^a^			
	*n*	OR	95% CI	*p* value	*n*	OR	95% CI	*p* value
*EuroQoL mobility*								
Clinical hip OA	216	5.49	(1.63, 18.5)	0.006	192	3.31	(0.37, 29.6)	0.284
Radiographic hip OA	192	2.05	(1.11, 3.79)	0.021	170	1.95	(0.90, 4.23)	0.089
Clinical knee OA	216	7.51	(3.56, 15.9)	< 0.001	192	6.05	(2.33, 15.71)	< 0.001
Radiographic knee OA	201	2.56	(1.36, 4.85)	0.004	179	2.55	(1.16, 5.58)	0.019
*EuroQoL self-care*								
Clinical hip OA	216	8.81	(2.67, 29.0)	< 0.001	192	7.13	(0.88, 57.8)	0.066
Radiographic hip OA	192	2.33	(0.94, 5.76)	0.066	170	2.02	(0.61, 6.68)	0.249
Clinical knee OA	216	9.52	(3.87, 23.4)	< 0.001	192	5.49	(1.67, 18.1)	0.005
Radiographic knee OA	201	2.81	(1.00, 7.90)	0.050	179	2.78	(0.69, 11.1)	0.148
*EuroQoL usual activities*								
Clinical hip OA	215	15.9	(3.40, 74.0)	< 0.001	191	19.6	(1.18, 326)	0.038
Radiographic hip OA	191	1.66	(0.88, 3.12)	0.117	169	1.29	(0.59, 2.86)	0.524
Clinical knee OA	215	9.20	(4.31, 19.7)	< 0.001	191	8.91	(3.45, 23.0)	< 0.001
Radiographic knee OA	200	3.25	(1.61, 6.54)	0.001	178	5.09	(1.99, 13.0)	0.001

^a^Adjusted for age, BMI, social class, smoker status, alcohol consumption, activity, baseline dietary calcium, years since menopause and HRT use

In general, men reported stronger associations between OA and moving around the house than women. In men there was a significant association with problems moving around inside and outside the house and a clinical diagnosis of OA at the hip or the knee (OR 14.4, 95% CI 2.98, 69.3, *p* = 0.001 and OR 7.85, 95% CI 2.90, 21.3, *p* < 0.001, respectively) and this remained robust following adjustment for confounders. Very similar associations with clinical hip and knee OA were seen when men were asked if they had difficulties mobilising around the kitchen specifically (OR 13.7, 95% CI 2.20, 85.6, *p* = 0.005 and OR 8.45, 95% CI 1.97, 36.2, *p* = 0.004, respectively) and these findings again remained significant following adjustment for confounders. Furthermore, clinical hip and knee OA in men were positively associated with problems accessing public facilities (OR 10.4, 95% CI 2.20, 49.5, *p* = 0.003 and OR 3.78, 95% CI 1.46, 9.80, *p* = 0.006, respectively) but only clinical hip OA remained significant following adjustment for confounders (OR 108, 95% CI 7.69, 1529, *p* = 0.001). Interestingly, following adjustment for confounders, knee OA was not associated with problems moving around the bathroom or toilet in men but significant associations were seen with both clinical hip OA and radiographic hip OA (bathroom: clinical hip OA—OR 290, 95% CI 11.1, 7559, *p* = 0.001 and radiographic hip OA—OR 7.71, 95% CI 1.28, 46.4, *p* = 0.026; toilet: clinical hip OA—OR 93.1, 95% CI 5.44, 1593, *p* = 0.002 and radiographic hip OA—OR 5.84, 95% CI 1.00, 34.1, *p* = 0.050) (Table 4).

**Table 4:** Hip and knee OA as explanatory variables for problems moving around in men

	Unadjusted				Adjusted^a^			
	*n*	OR	95% CI	*p* value	*n*	OR	95% CI	*p* value
*Problems moving around inside and outside house*								
Clinical hip OA	219	14.4	(2.98, 69.3)	0.001	192	49.0	(4.75, 506)	0.001
Radiographic hip OA	201	1.18	(0.47, 2.97)	0.725	176	1.70	(0.54, 5.33)	0.360
Clinical knee OA	216	7.85	(2.90, 21.3)	< 0.001	188	7.97	(2.12, 30.0)	0.002
Radiographic knee OA	201	1.79	(0.67, 4.76)	0.242	175	0.77	(0.23, 2.60)	0.674
*Problems moving around bathroom*								
Clinical hip OA	219	34.0	(6.50, 178)	< 0.001	192	290	(11.1, 7559)	0.001
Radiographic hip OA	201	2.85	(0.72, 11.4)	0.138	176	7.71	(1.28, 46.4)	0.026
Clinical knee OA	216	6.22	(1.81, 21.4)	0.004	188	4.72	(0.96, 23.1)	0.056
Radiographic knee OA	201	1.52	(0.41, 5.54)	0.530	175	0.88	(0.19, 4.09)	0.868
*Problems moving around kitchen*								
Clinical hip OA	219	13.7	(2.20, 85.6)	0.005	192	63.5	(2.81, 1434)	0.009
Radiographic hip OA	201	1.57	(0.34, 7.22)	0.560	176	3.31	(0.54, 20.3)	0.196
Clinical knee OA	216	8.45	(1.97, 36.2)	0.004	188	12.1	(1.41, 104)	0.023
Radiographic knee OA	201	2.55	(0.48, 13.5)	0.270	175	1.56	(0.25, 9.93)	0.636
*Problems moving around toilet*								
Clinical hip OA	219	22.0	(4.11, 117)	< 0.001	192	93.1	(5.44, 1593)	0.002
Radiographic hip OA	201	2.41	(0.59, 9.93)	0.222	176	5.84	(1.00, 34.1)	0.050
Clinical knee OA	216	5.58	(1.46, 21.3)	0.012	188	4.17	(0.72, 24.2)	0.112
Radiographic knee OA	201	2.04	(0.50, 8.40)	0.323	175	1.72	(0.33, 9.14)	0.522
*Problems accessing public facilities* ^b^								
Clinical hip OA	219	10.4	(2.20, 49.5)	0.003	192	108	(7.69, 1529)	0.001
Radiographic hip OA	201	0.44	(0.17, 1.10)	0.079	176	0.41	(0.11, 1.55)	0.190
Clinical knee OA	216	3.78	(1.46, 9.80)	0.006	188	2.07	(0.56, 7.61)	0.274
Radiographic knee OA	201	1.20	(0.51, 2.81)	0.683	175	0.44	(0.12, 1.60)	0.212

^a^Adjusted for age, BMI, social class, smoker status, alcohol consumption, activity and baseline dietary calcium

^b^Problems with accessing public facilities such as grocery shops, bus stops or banks

Overall, the associations between OA and difficulties with reported mobility were weaker in women. In contrast to men there was no association between either hip or knee OA and difficulties accessing public facilities and moving around the bathroom following adjustment for confounders. Knee, but not hip, OA in women was associated with general problems moving inside and outside the house following adjustment for confounders (clinical knee OA: OR 13.5, 95% CI 4.09, 44.7, *p* < 0.001; radiographic knee OA: OR 12.4, 95% CI 2.41, 64.0, *p* = 0.003) and problems moving around the kitchen after adjustment for confounders (clinical knee OA: OR 5.38, 95% CI 1.17, 24.7, *p* < 0.030). Similarly to men, clinical hip OA was associated with problems moving around the toilet (OR 12.1, 95% CI 3.34, 43.6, *p* < 0.001) and this association also remained significant post adjustment for confounders (Table 5).

**Table 5:** Hip and knee OA as explanatory variables for problems moving around in women

	Unadjusted				Adjusted^a^			
	*n*	OR	95% CI	*p* value	*n*	OR	95% CI	*p* value
*Problems moving around inside and outside house*								
Clinical hip OA	216	6.73	(2.11, 21.4)	0.001	192	5.32	(0.58, 48.8)	0.139
Radiographic hip OA	192	1.84	(0.87, 3.88)	0.110	170	2.43	(0.82, 7.18)	0.108
Clinical knee OA	216	14.7	(6.45, 33.5)	< 0.001	192	13.5	(4.09, 44.7)	< 0.001
Radiographic knee OA	201	5.32	(1.97, 14.4)	0.001	179	12.4	(2.41, 64.0)	0.003
*Problems moving around bathroom*								
Clinical hip OA	216	6.84	(2.01, 23.2)	0.002	192	7.85	(0.75, 82.2)	0.085
Radiographic hip OA	192	1.70	(0.71, 4.08)	0.233	170	2.20	(0.49, 9.83)	0.303
Clinical knee OA	216	7.63	(2.95, 19.7)	< 0.001	192	3.49	(0.82, 14.8)	0.090
Radiographic knee OA	201	5.44	(1.56, 19.0)	0.008	179	4.00	(0.66, 24.2)	0.130
*Problems moving around kitchen*								
Clinical hip OA	216	7.30	(2.14, 25.0)	0.002	192	7.21	(0.59, 87.8)	0.121
Radiographic hip OA	192	1.71	(0.69, 4.24)	0.249	170	3.19	(0.53, 19.1)	0.204
Clinical knee OA	216	11.1	(4.09, 30.4)	< 0.001	192	5.38	(1.17, 24.7)	0.030
Radiographic knee OA	201	4.80	(1.37, 16.9)	0.014	179	3.37	(0.47, 24.2)	0.227
*Problems moving around toilet*								
Clinical hip OA	216	12.1	(3.34, 43.6)	< 0.001	192	15.7	(1.36, 180)	0.027
Radiographic hip OA	192	1.75	(0.61, 5.03)	0.301	170	2.09	(0.44, 9.80)	0.351
Clinical knee OA	216	13.8	(4.07, 46.8)	< 0.001	192	5.00	(0.96, 26.1)	0.056
Radiographic knee OA	201	5.01	(1.10, 22.9)	0.037	179	5.24	(0.49, 56.0)	0.171
*Problems accessing public facilities* ^b^								
Clinical hip OA	216	6.39	(1.98, 20.6)	0.002	192	1.51	(0.11, 20.2)	0.756
Radiographic hip OA	192	2.02	(0.89, 4.60)	0.093	170	2.23	(0.58, 8.54)	0.242
Clinical knee OA	216	7.98	(3.43, 18.6)	< 0.001	192	3.24	(0.76, 13.9)	0.113
Radiographic knee OA	201	2.22	(0.89, 5.51)	0.087	179	1.22	(0.32, 4.65)	0.770

^a^Adjusted for age, BMI, social class, smoker status, alcohol consumption, activity, baseline dietary calcium, years since menopause and HRT use

^b^Problems with accessing public facilities such as grocery shops, bus stops or banks

## Discussion

In the current study, we have shown that a diagnosis of lower limb OA is strongly related to the ability to undertake ADL in older adults. We have demonstrated that in both men and women a clinical diagnosis of hip or of knee OA and a radiological diagnosis of knee OA in women is associated with difficulties in mobility, ability to self-care and performing usual activities. Our data, therefore. suggests that using a clinical criteria to diagnose OA, especially in men, is more sensitive at identifying individuals who are at risk of functional impairment then employing a radiographic diagnosis alone. This is consistent with previous studies that have shown radiographic knee OA correlates poorly with the physical symptoms of OA [22]. Indeed, a previous study utilising participants from the HCS demonstrated that a substantial proportion of men and women who were diagnosed with radiographic OA did not have self-reported or diagnosis of clinical OA (57.7%) [7]. Our study therefore lends further credence to the argument that in the clinical setting where the focus of intervention is on the improvement of symptoms, the use of a clinical definition of knee OA, which includes pain, may be more useful than relying on a radiographic diagnosis alone to identify individuals at risk of functional impairment and target resources accordingly. Indeed, the International Rheumatologic Board (IRB) recently proposed guidelines for the diagnosis of OA in primary care which are based on the ACR criteria for clinical OA [23].

Our data are consistent with previous studies that have shown that OA leads to impairments in quality of life and ADL and as a consequence, results in dependency, institutionalisation and increased health-care costs [24, 25]. The majority of these studies have been qualitative in nature but a recent study by Stamm and colleagues explored the limitations in the ADLs in older adults in a population-based survey of 3097 subjects aged ≥ 65 years in Austria. They demonstrated that OA was associated with a with a 68% higher chance of impairment of intense ADLs such as lifting and carrying a shopping bag of over 5 kg of weight, bending and kneeling down, walking 500 m without the use of aids, climbing stairs without the use of aids and heavy housework [25].

We have observed in the current study that problems with mobilising around specific rooms in the house and accessing public facilities varied according to site and sex. The ability of men to mobilise around the kitchen and to access public facilities was impeded by both hip and knee OA with stronger associations seen when a clinical definition of OA is utilised. In contrast to men, there was no significant association between lower limb OA and difficulties accessing public facilities or mobilising around the bathroom in women following adjustment for confounders. In both sexes, however, hip OA was associated with difficulties mobilising around the toilet. These results suggest that the specific movements required for mobilising around a toilet, which is usually in a confined space, and the action of getting on and off a toilet are more impeded by hip OA than knee OA. Conversely, mobilising around traditionally larger rooms, such as the kitchen, appears to be more impeded by knee OA. These results are particularly pertinent when considering the benefit of potentially tailoring occupational therapy (OT) services to different groups of patients and that we should ask about ADL in the clinic setting. Indeed, there is strong evidence to suggest that to successfully design healthcare programs for the treatment of OA it is essential to consider what patients need and prefer, and how they value various aspects of a health intervention [26].

Our study is limited in that the results may not be entirely representative of the wider UK population since all recruited participants were born in the county of Hertfordshire and at age 75 were still living there (as had been the case in previous studies). We have demonstrated that this cohort are a good representation of the general population with regard to body build and lifestyle factors, such as smoking and alcohol intake, therefore suggesting that selection bias was minimal [27]. Furthermore, all comparisons undertaken were internal. There is a possibility that there may have been some inconsistencies in the interpretation of radiographs. To minimise this risk, however, two experienced rheumatologists were used to grade the radiographs, with high inter-observer concordance. We have also previously shown that for both clinical and radiographic assessment of OA used within this current study good levels of agreement exists between- and within-observer variation. Briefly, repeatability for all observations was graded either good or excellent by multiple observers [28]. Additionally, only a small number of participants (7 men and 13 women) fit the diagnostic criteria for clinical hip OA which may limit our power to detect statistically significant relationships. Finally, the study population did not specifically exclude individuals who were awaiting a joint replacement operation and therefore some of these individuals may have been included in the analysis. There is evidence to suggest, however, that although functional improvements following knee arthroplasty are excellent regardless of age, knee arthroplasty contributes little to the quality of life in older patients (octogenarians) [29].

## Conclusions

Our study shows that a diagnosis of OA is strongly related to the ability to undertake ADL in older adults. Limitations in ADLs and mobility vary according to site and sex and these differences should be considered in the clinical setting. These data support the requirement for functional assessment and corresponding interventions to prevent worsening functional decline in individuals with OA and the consequent health and social problems which would arise at great expense to the individual and society.
